# HSV-tk/GCV gene therapy mediated by EBV-LMP1 for EBV-associated cancer

**DOI:** 10.1186/1756-9966-27-42

**Published:** 2008-09-23

**Authors:** Yang Lifang, Tang Min, Ai Midan, Cao Ya

**Affiliations:** 1 Molecular Biology Research Center, Cancer Research Institute, XiangYa School of Medicine, Central South University, ChangSha, Hunan, 410078, PR China

## Abstract

**Background:**

To investigate the feasibility of gene therapy in treating Epstein-Barr virus (EBV)-associated cancer by employing the suicide gene, herpes simplex virus thymidine kinase/ganciclovir (HSV-tk/GCV), which uses the signaling pathway through the HIV-long terminal repeat (LTR) gene which is expressed from a nuclear factor-κB (NF-κB)-binding motif-containing promoter that is regulated by EBV-latent membrane protein 1 (LMP1) via NF-κB.

**Methods:**

First, we constructed the plasmid pVLTR-tk, which was regulated by EBV-LMP1 via NF-κB, and then investigated the cytotoxic effect of the pVLTR-tk/GCV on cancer cells, using MTT assays, clonogenic assays, flow cytometry, and animal experiments.

**Results:**

The activation of TK was increased after transfection of the pVLTR-tk into the EBV-LMP1 positive cells. After GCV treatment, the clonogenicity and survival of the cells substantially declined, and a bystander effect was also observed. The LMP1 positive cells exhibited remarkable apoptosis following pVLTR-tk/GCV treatment, and the pVLTR-tk/GCV restrained tumor growth in vivo for EBV-LMP1 positive cancers.

**Conclusion:**

The pVLTR-tk/GCV suicide gene system may be used as a new gene targeting strategy for EBV-associated cancer.

## Background

The Epstein-Barr virus (EBV) has been implicated in the pathogenesis of several human malignant tumors including nasopharyngeal carcinoma (NPC), Hodgkin's disease, and Burkitt's lymphoma [1]. EBV-encoded latent membrane protein 1 (LMP1), which is considered an oncoprotein, plays an important role in the progress of carcinogenesis via regulation of transformation, proliferation, apoptosis, and other biological processes of the cell [2]. EBV-LMP1 has also been found to regulate the development and progression of tumors via activated nuclear factor-κB (NF-κB) [3]. Our research has previously showed that EBV-LMP1 continuously activates NF-κB by directly combining tumor necrosis factor receptor-activated factor (TRAF) with the C-terminal activating regions 1 and 2 (CTAR1 and CTAR2) of LMP1 [4-6].

The long terminal repeat (LTR) of HIV is located at both the N-terminal and C-terminal domains of the HIV genome. Many *cis*-acting elements are associated with the expression of charge of the HIV gene in the LTR, including two NF-κB motifs. Several studies have indicated that LMP1 possesses the ability to transactivate HIV-LTR through the NF-κB binding motifs [7-9].

The herpes simplex virus-thymidine kinase (HSV-tk) gene has been used in the treatment of many different types of tumors in clinical trials [10,11]. Research has showed that HSV-tk/GCV exhibits not only direct cytotoxicity but also a bystander effect (BSE), which builds up effectively the function of killing and wounding of the HSV-tk/GCV system [12,13]. However, one of the major limitations of gene therapy using HSV-tk/GCV is the effective expression of therapeutic genes in target cells.

In this study, we examined the feasibility of using the signaling pathway of LMP1, which stimulates HIV-LTR via the two NF-κB binding sites, as a therapeutic strategy for the treatment of EBV-associated cancer. Because LMP1 is expressed only in tumor cells (and not in normal tissue cells), this therapeutic strategy could enhance the tumor-specific targeting capacity of HSV-tk/GCV for EBV-associated cancer.

## Methods

### Cells

CNE1: The LMP1 negative high differentiated nasopharyngeal squamous carcinoma cell line; CNE1-LMP1: stable LMP1 integrated nasopharyngeal squamous carcinoma cell line; HNE2-LMP1: lowly differentiated and stable LMP1 integrated nasopharyngeal squamous carcinoma cell line [14,15]; Raji (ATCC CCL 86): Burkitt's lymphoma cell line, LMP1 positive cell; and B95-8(ATCC CRL 1612): B lymphocyte cell line transformed by the EBV. Cells were cultured in RPMI 1640 medium (GIBCO) supplemented with 10% fetal bovine serum.

### Plasmids

The HSV-TK fragment from the HSV-TK gene-containing plasmid, pAB109, was excised by *Bgl*II/*Pvu*II, and subcloned into the pGN-LTR vector (the LacZ reporter plasmid regulated by HIV-LTR, Dr. Chang YS, Chang-Gung University) at the *Eco*RI/*Sma*I site (with LacZ removed). This is the expression plasmid that contains HSV-tk regulated by HIV-LTR. The plasmid constructs were verified by sequence analysis.

### Assay of TK activity

Forty-eight hours after NPC cells (CNE1, CNE1-LMP1, HNE2-LMP1) were transiently transfected using the SuperFect™ transfection reagent (QIAGEN) with pVLTR-tk, cultures were harvested and lysed with lysis buffer (50 mM Tris-HCl, 1 mM EDTA, 20 g/L sodium dodecyl sulfate (SDS), 5 mM DTT, and 10 mM phenylmethylsulfonyl fluoride). Following centrifugation, 20 μL of supernatant was mixed with 100 μL of TK reaction buffer (125 mM Tris-HCl, pH 7.8; 2 mM MgCl_2_; 200 mM KCl; 100 mM NH_4_Cl; 4 mM mercaptoethanol; 2 mM ATP; 0.5 mg/mL fetal bovine serum; and 3 × 10^-7^M ^3^H thymidine), and incubated at 37°C for 30 min. After incubation, 50 μL of the reaction mixture was spotted onto a piece of 1 cm^2 ^Whatman DE-81 filter paper. The filters were washed three times with 95% ethanol, transferred to 5 mL of scintillation buffer after drying, and counted using the Beckman LS 5000 TD multipurpose scintillation counter (Beckman Instruments). The efficiency of transfection proofread by co-transfection of pRSVβ-gal Reporter System (Promega).

### Cell proliferation assay

Cells were plated at a density of 2 × 10^4 ^cells/well in 48-well plates 24 hours before transfection with the pVLTR-tk using the SuperFect™ transfection reagent. Following the 24-hours incubation period, cells were cultured for 48 hours in the presence of different concentrations (50 μg/mL, 100 μg/mL, 200 μg/mL, 300 μg/mL, 500 μg/mL) of GCV. At the end of the culture period, 40 μL of MTT (5 mg/mL) was added to each well. After 4 hours of incubation with MTT, DMSO (200 μL/well) was added. The OD value of each well was read using a microplate reader (ELX 800; Bio-tek Instruments) at 570 nm. The survival rate of cells was expressed as *A/B 100%*, where *A *was the absorbance value from the experimental cells and *B *was that from the control cells, which were not treated with GCV.

### Bystander effect analysis

Twenty-four hours after tumor cells were transfected with the pVLTR-tk/GCV, different proportions (0%, 10%, 50%, 90%, and 100%), respectively, of transfected and untransfected cells that had been adjusted to a density of 2 × 10^4 ^cells/well were cocultured in 48-well plates at 37°C for 48 hours in the presence of 200 μg/mL of GCV. The bystander effect was determined using the MTT method, as described previously.

### Clonogenic assay

Cells (5 × 10^2 ^cells/well) were transfected with the pVLTR-tk in the presence of 200 μg/mL of GCV. When the resistant colonies were an appropriate size following incubation, the plates were fixed in 10% methanol and 5% acetic acid for 10 min. After the plates were stained in 0.1% crystal violet/20% ethanol for 5 min, the colonies were counted.

### Detection of apoptosis by flow cytometry

Forty-eight hours after transfection of the pVLTR-tk using the SuperFect™ transfection reagent into 1 × 10^6 ^cells and treatment with 200 μg/mL of GCV, both adherent and floating cells were harvested by trypsinization. The cells were centrifuged, and the cell pellets were washed with phosphate-buffered saline (PBS). The cells were fixed in 70% ethanol overnight. They were then washed and resuspended in Hank's buffered saline solution, and incubated in phosphate-citric acid buffer for 5 min. Following the 5-min incubation period, the cells were centrifuged, and the pellet was resuspended in Hank's buffered saline solution containing propidium iodide (10 μg/mL) and RNase (100 μg/mL). Following the 30-min incubation period at room temperature, the cells were quantified with a FACScan (Becton Dickinson) flow cytometer, using 488- and 630-nm filters for excitation and emission, respectively. The salmon sperm DNA (ssDNA) used as a DNA toxicity control.

### Animal experiment

Twenty-four hours after transfection with pVLTR-tk using SuperFect™ transfection reagent, 5 × 10^6 ^cancer cells (CNE1, CNE1-LMP1) in 0.2 mL of serum-free RPMI 1640 medium were inoculated s.c. into the right flank of 8- to 12-week-old male BALB/c nude mice (4-5 mice for each treatment group). The cancer growth was monitored every 2 days using calipers. When tumors reached a mean tumor diameter of 4 to 5 mm, GCV (100 μmg/g avoirdupois) was injected directly into the tumor every 24 hours for 7 days. Following a few days of observation, the mice were sacrificed by cervical dislocation, tumors were removed and frozen rapidly, and 5.0-μm cryosections were prepared for histopathological studies.

### Histopathological examination

The cryosections from the treated tumor tissues were stained with hematoxylin and eosin, and analyzed by light microscopy.

### Statistical analysis

All the data were analyzed using the Student's *t *test. The statistical difference *P *< 0.05 was considered significant.

## Results

### EBV-LMP1 promote the TK activity of the pVLTR-tk

**Figure 1:**
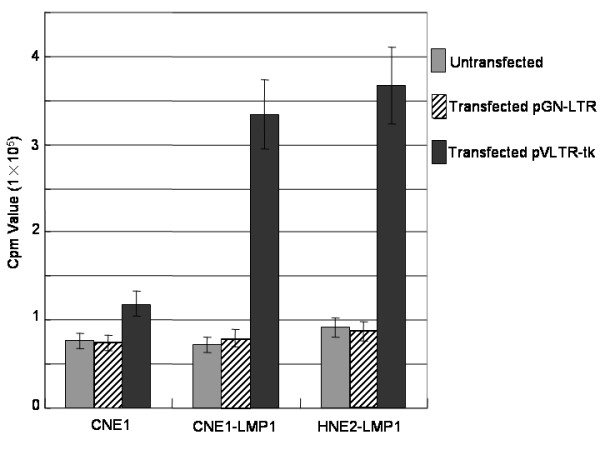
**Thymidine kinase (tk) activity assay.** Tk activity of CNE1, CNE1-LMP1, and HNE2-LMP1 cells after transfection with pVLTR-tk.

As shown in Figure 1, the TK enzymatic activity of CNE1 cells transfected with the pVLTR-tk was similar to that of untransfected cells, with the cpm volume being about 1 × 10^5^, indicating that the expression of the endogenous tk gene was at a low level. However, unlike the untransfected cells, the TK enzymatic activity in CNE1-LMP1 and HNE2-LMP1 cells transfected with the pVLTR-tk maintained high levels-about three times higher than that in CNE1 cells (*P *< 0.01)-indicating that high expression of the pVLTR-tk gene is regulated by EBV-LMP1.

### Inhibition of LMP1 positive cells growth with pVLTR-tk/GCV

**Figure 2:**
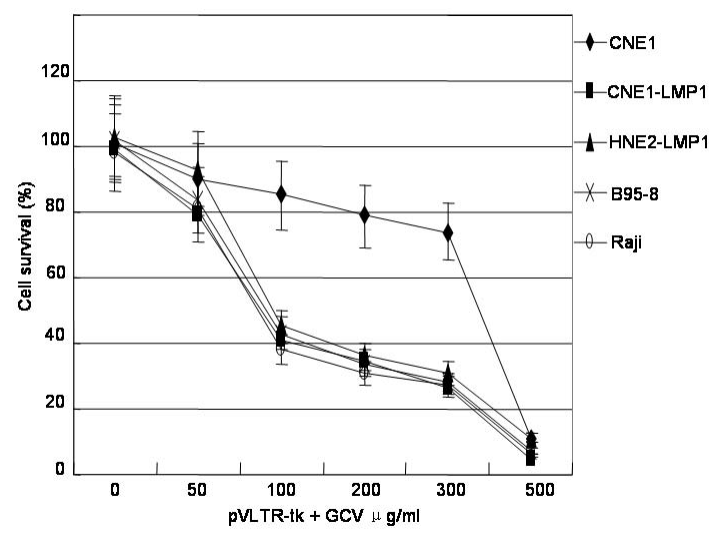
**pVLTR-tk/GCV inhibit the cell proliferation in LMP1 positive cells.** Cells were transfected with pVLTR-tk and treated with GCV. Cell proliferation was measured by MTT analysis.

CNE1, CNE1-LMP1, HNE2-LMP1, B95-8, and Raji cells were transfected with the pVLTR-tk, and incubated with GCV at various concentrations. A decrease in cell viability was observed upon increasing GCV concentration, the highest toxicity being obtained at 500 μg/mL of GCV for all cell lines. These results suggested that for GCV concentrations higher than 500 μg/mL, the limiting factor affecting cytotoxicity is not related to the level of thymidine kinase expression. As illustrated in Figure 2, in CNE1-LMP1, HNE2-LMP1, B95-8, and Raji cells, the survival cell rates were about 80% with 50 μg/mL of GCV, about 40% with 100 μg/mL and 200 μg/mL of GCV, and about 20% with 300 μg/mL of GCV. When the effects at the same concentration of GCV are compared, these cells seem to be more sensitive to GCV than CNE1 cells. (The level of toxicity remained unchanged in CNE1 cells at 50 to 300 μg/mL of GCV.) These results indicated that transfection with the pVLTR-tk increases sensitivity to GCV in EBV-LMP1 positive cells.

### A bystander effect was found in the presence of pVLTR-tk/GCV

**Figure 3:**
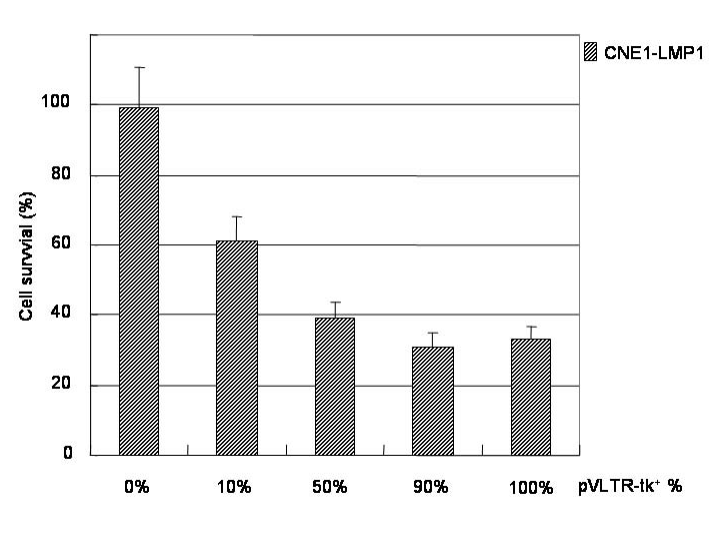
A bystander effect was found in the presence of pVLTR-tk.

As shown in Figure 3, 50% of these transfectants could kill 60% of the cocultured cells, and 10% of these transfectants could kill 40% of the cocultured cells, which is indicative of a marked bystander effect. However, the most prominent bystander effect was found in the presence of the pVLTR-tk.

### pVLTR-tk/GCV inhibit survival of the LMP1 positive cells

**Figure 4:**
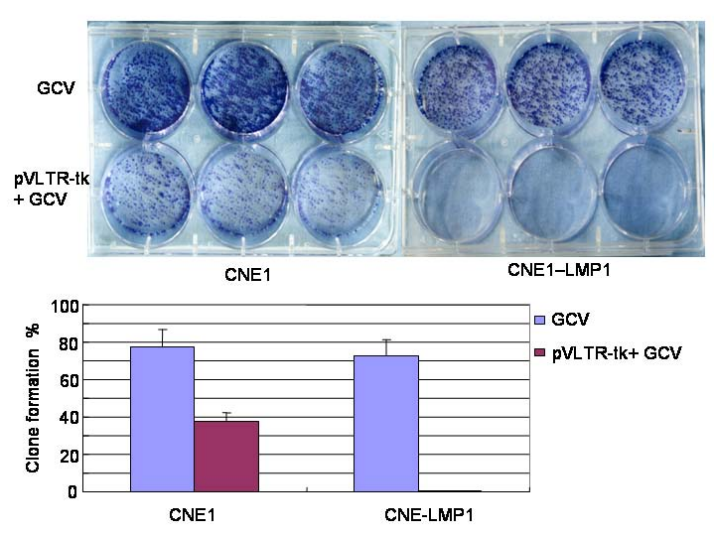
**pVLTR-tk/GCV restrain cell survival in LMP1 positive cells.** Cells were transfected with pVLTR-tk and treated with GCV. Cell survival was measured by a Clonogenic assay.

As shown in Figure 4, the average cell survival rate of CNE1 cells treated with GCV (77.4%) was higher than that of CNE1 cells transfected with pVLTR-tk and treated with GCV (37.6%). The difference in the average cell survival rate between CNE1-LMP1 cells treated with GCV (72.8%) and that of CNE1-LMP1 cells transfected with pVLTR-tk and treated with GCV (0.5%, *P *< 0.001) was even more pronounced. The results indicated that EBV-LMP1 positive cells are more sensitive to pVLTR-tk/GCV in clonogenic assays.

### Induce the apoptosis in LMP1 positive cells with pVLTR-tk/GCV

**Figure 5:**
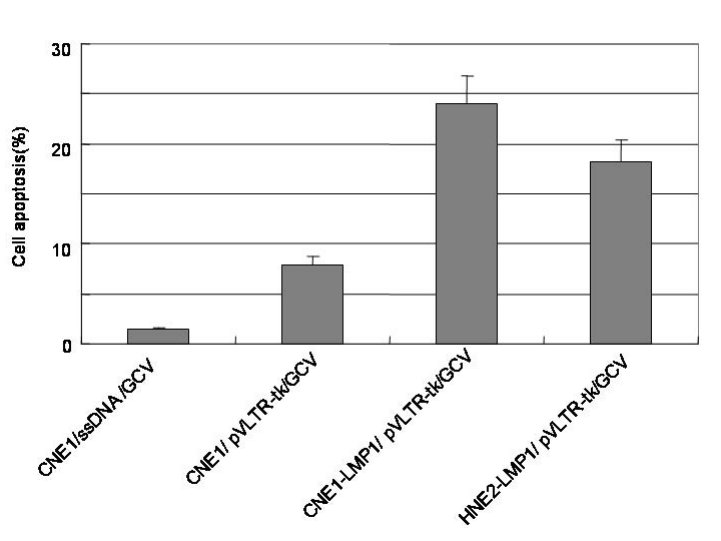
**pVLTR-tk/GCV enhance apoptosis in LMP1 positive cells. **Cells were transfected with pVLTR-tk and treated with GCV. Cell apoptosis was measured by flow cytometry.

The percentage of cell apoptosis in HNE2-LMP1 and CNE1-LMP1 cells was about 20% following treatment with the pVLTR-tk/GCV. A slightly higher percentage of apoptosis occurred in CNE1 cells (7.8%, *P *< 0.01) compared with that of CNE1 cells transfected with ssDNA/GCV (1.4%) (Figure 5). The results indicated that treatment of EBV-LMP1 positive cells with pVLTR-tk/GCV induces cell apoptosis.

### pVLTR-tk/GCV restrain the tumor growth

**Figure 6:**
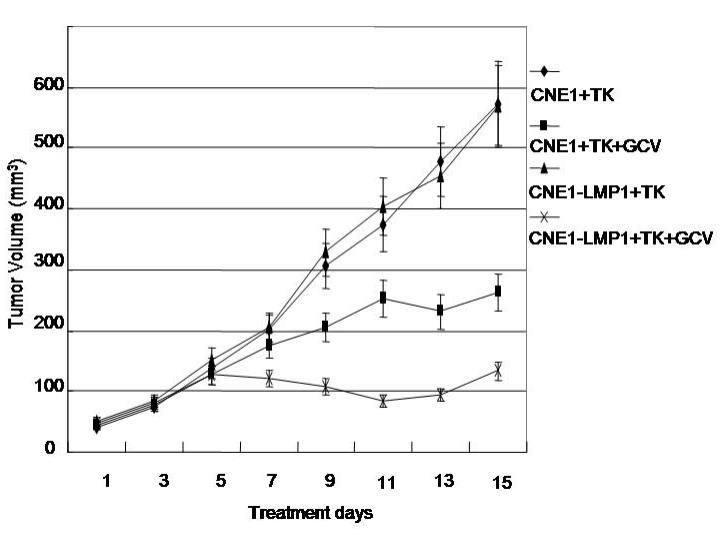
**Inhibition of nasopharyngeal carcinoma growth in athymic nude mice with pVLTR-tk/GCV treatment.** Five mice per group were injected intratumorally every 24 hours for 7 days with GCV (100 μmg/g avoirdupois). The cancer growth was monitored every 2 days using calipers.

As shown in Figure 6, treatment with the pVLTR-tk/GCV significantly suppressed the growth of EBV-LMP1 s.c. tumors when compared with that of CNE1 cells (*P *< 0.01), although EBV-LMP1 negative CNE1 s.c. tumors also exhibited a moderate restrain effect after treatment with pVLTR-tk/GCV. These results suggested that the pVLTR-tk/GCV has a favorable antitumor effect in vivo.

### EBV-LMP1 is capable of enhancing the cytotoxic effect of pVLTR-tk/GCV

**Figure 7:**
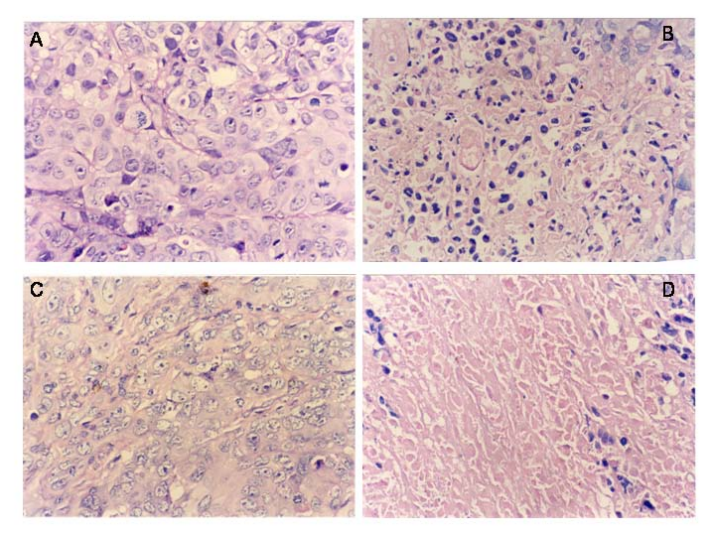
**Histopathological analysis of tumor tissues after pVLTR-tk/GCV treatment. **A: CNE1+TK (400×); B: CNE1+TK+GCV (400×); C: CNE1-LMP1+TK (400×); D: CNE1-LMP1+TK+GCV (400×).

Hematoxylin and eosin staining showed that the tumor status of the CNE1+TK group and that of the CNE1-LMP1+TK group were concordant with the pathology of the tissue. In the CNE1+TK+GCV group, there were some putrescence cells, whereas in the CNE1-LMP1+TK+GCV group, there were a great number of putrescence cells. Therefore, EBV-LMP1 is capable of enhancing the cytotoxic effect of pVLTR-tk/GCV (Figure 7).

## Discussion

HSV-tk/GCV systems have been shown to induce effectively the killing and wounding of many malignant tumors [16,17]. However, the application of HSV-tk is blocked badly because of the lack of tumor-specific targeting capability; thus, the key to improve this type of therapy is finding a way to improve tumor specificity [18,19].

Previous studies have indicated that LMP1 possesses the ability to transactivate HIV-LTR through the NF-κB binding motifs. Chang's data showed that the HSV-tk gene expressed from a NF-kappa B-binding motif-containing promoter that is regulated by LMP1 could inhibit cell proliferation in LMP1 positive cells such as C33A cell clones and restrain the tumor growth in vivo[9]. In this study, the effects of the pVLTR-tk, which expresses HSV-tk that is regulated by EBV-LMP1 via NF-κB was investigated by employing different cell lines, including the CNE1, CNE1-LMP1, HNE2-LMP1, and other EBV-related cancer cells. The result showed that the pVTR-TK/GCV system had toxicity to the EBV-related cells and tumor. At the same time, we also observed a significant bystander effect. In addition, ours results indicated that apoptosis is the main mechanism involved in the death of LMP1 positive cells upon their treatment with the pVLTR-tk/GCV system. In conclusion, the combination of pVLTR-tk gene expression and treatment with GCV could result in the capacity to kill LMP1-expressing tumor cells effectively.

In vitro and in vivo experiments in the present study revealed that EBV-LMP1 negative CNE1 cells also exhibited a moderate killing effect after treatment with pVLTR-tk/GCV. It will be important to investigate the toxic effects of pVLTR-tk/GCV genes in normal tissues. However, this new targeted strategy of the HSV-tk/GCV system may be used as a gene therapy strategy for EBV-LMP1 positive cancers. Because LMP1 is expressed only in tumor cells, and not in normal tissue cells, this therapeutic strategy could enhance the targeting efficacy of the HSV-tk/GCV system.

## Competing interests

The authors declare that they have no competing interests.

## Authors' contributions

YL carried out the molecular biologic studies, animal experiment, participated in the cell biologic studies and drafted the manuscript. TM carried out the cell biologic studies. AM carried out the assay of TK enzyme activity. CY conceived of the study, and participated in its design and coordination. All authors read and approved the final manuscript.
